# Functional Refinement in the Projection from Ventral Cochlear Nucleus to Lateral Superior Olive Precedes Hearing Onset in Rat

**DOI:** 10.1371/journal.pone.0020756

**Published:** 2011-06-09

**Authors:** Daniel T. Case, Xiwu Zhao, Deda C. Gillespie

**Affiliations:** 1 Neuroscience Graduate Program, McMaster University, Hamilton, Ontario, Canada; 2 Department of Psychology, Neuroscience & Behaviour, McMaster University, Hamilton, Ontario, Canada; The Research Center of Neurobiology-Neurophysiology of Marseille, France

## Abstract

Principal neurons of the lateral superior olive (LSO) compute the interaural intensity differences necessary for localizing high-frequency sounds. To perform this computation, the LSO requires precisely tuned, converging excitatory and inhibitory inputs that are driven by the two ears and that are matched for stimulus frequency. In rodents, the inhibitory inputs, which arise from the medial nucleus of the trapezoid body (MNTB), undergo extensive functional refinement during the first postnatal week. Similar functional refinement of the ascending excitatory pathway, which arises in the anteroventral cochlear nucleus (AVCN), has been assumed but has not been well studied. Using whole-cell voltage clamp in acute brainstem slices of neonatal rats, we examined developmental changes in input strength and pre- and post-synaptic properties of the VCN-LSO pathway. A key question was whether functional refinement in one of the two major input pathways might precede and then guide refinement in the opposite pathway. We find that elimination and strengthening of VCN inputs to the LSO occurs over a similar period to that seen for the ascending inhibitory (MNTB-LSO) pathway. During this period, the fractional contribution provided by NMDA receptors (NMDARs) declines while the contribution from AMPA receptors (AMPARs) increases. In the NMDAR-mediated response, GluN2B-containing NMDARs predominate in the first postnatal week and decline sharply thereafter. Finally, the progressive decrease in paired-pulse depression between birth and hearing onset allows these synapses to follow progressively higher frequencies. Our data are consistent with a model in which the excitatory and inhibitory projections to LSO are functionally refined in parallel during the first postnatal week, and they further suggest that GluN2B-containing NMDARs may mediate early refinement in the VCN-LSO pathway.

## Introduction

The superior olivary complex in the auditory brainstem includes the key nuclei responsible for azimuthal sound localization. In particular, the lateral superior olive (LSO) compares converging excitatory and inhibitory inputs to compute sound level differences between the two ears for high frequency sounds [1], [2]. This computation requires that the LSO be precisely tonotopically organized such that the excitatory and inhibitory inputs converge to the same isofrequency band (for review, see [3]). The excitatory, glutamatergic projection arises in the ipsilateral anteroventral cochlear nucleus (AVCN) [4],[5], whereas the inhibitory, glycinergic projection arises in the ipsilateral medial nucleus of the trapezoid body (MNTB), a sign-inverting nucleus driven by the contralateral VCN [6]–[9]. A question of fundamental importance is how these primary inputs of opposing sign are coordinately refined during development to provide the finescale tuning necessary for computing interaural level differences in the adult LSO (for reviews, see [10], [11]).

Axons of both excitatory and inhibitory projections can be detected in the LSO shortly before birth in the rat [12]. These early inputs, which are functional but weak [13], undergo developmental refinement that results in frequency-matched excitation and inhibition [14], [15]. Of these two pathways, relatively more is known about development of the inhibitory projection. Patterned, spontaneous activity from the cochlea [16]–[19], which increases steadily from around postnatal day 3 (P3) to hearing onset at P12 [20], is thought to direct activity-dependent refinement in the MNTB-LSO pathway [21]. Functional refinement of the rat MNTB-LSO pathway is accompanied by changes in quantal size and quantal content and is mediated by largely unknown mechanisms of plasticity that may require glutamate release from MNTB terminals and that occur primarily between P3 and P8 [22]–[25]. Although it has often been assumed that during this same period the ipsilateral VCN inputs to the LSO undergo developmental refinement, in fact much less is known about how the VCN-LSO pathway normally develops.

Ipsilateral VCN afferents invade the LSO at around embryonic day 18 in rat and are excitatory onto LSO neurons within 2 days [12], [13]. AMPA receptors (AMPARs), NMDA receptors (NMDARs) and metabotropic glutamate receptors (mGluRs) are all expressed in LSO neurons during early postnatal development [26], [27], and calcium-imaging studies have shown AMPARs, NMDARs and mGluRs to be activated in the VCN-LSO pathway in the first postnatal week in mouse [28]. Neither functional refinement nor in vitro synaptic plasticity has yet been reported in this pathway, however, and central aspects of pathway development remain to be determined, such as whether, to what degree, and when functional and morphological refinement occur.

Because the LSO's two major input projections must map onto the same space in tonotopic register with one another, an important first question is to understand whether one of the two inputs is initially established and refined—later acting as a template for the opposite projection—or whether both inputs are established and refined relatively independently. In order to better understand the mechanisms that may govern circuit refinement and tonotopic alignment of excitatory and inhibitory inputs, we have examined developmental changes in several synaptic properties in the VCN-LSO pathway, including measures of input strength, receptor types and subunits, and properties of neurotransmitter release. We find an apparent decrease in input number, and increase in input strength, during a period that parallels that of functional refinement in the MNTB-LSO pathway. We further find that NMDAR-mediated signaling is elevated during the period of functional refinement, and that a GluN2B component is prevalent in the NMDAR-mediated response before P8 and declines thereafter. As the timecourse of refinement and the activation of GluN2B-containing NMDARs in the VCN-LSO pathway closely shadow similar events in the developing MNTB-LSO pathway, our results support a model in which the excitatory and inhibitory inputs to LSO neurons are refined over the same time frame, presumably independently, and likely using some of the same synaptic molecules and mechanisms.

## Materials and Methods

### Ethics Statement

All procedures adhered to Canadian Council on Animal Care guidelines and were approved by the Animal Research Ethics Board of McMaster University (Permit #08-08-36).

### Slice preparation and physiology

Sprague-Dawley rats (Charles-River Laboratories) aged postnatal days 1 to 12 (P1-12; P0 is day of birth) were anaesthetized with isofluorane and quickly decapitated, and the brains were removed into ice-cold artificial cerebrospinal fluid (ACSF, pH 7.2) containing (in mM): 125 NaCl, 1 MgSO_4_, 5 KCl, 1.25 KH_2_PO_4_, 10 dextrose, 26 NaHCO_3_, 2 CaCl_2_•2H_2_O, 1 kynurenic acid. The brainstem was cut at 300 µm (Vibratome 3000 Series), and slices containing the LSO were transferred to a humidified interface chamber where they were allowed to recover for at least 1 hour at room temperature (∼20C). For recording, slices were transferred to a recording chamber at an upright microscope. Slices were kept at room temperature and continuously perfused with ACSF superfused with 95% O_2_/5% CO_2_. Strychnine (1 µM) and picrotoxin (50 µM) were added to the perfusate to block glycine and GABA_A_ receptors, respectively; kynurenic acid was not added to ACSF used in recordings. For experiments performed in Mg^++^-free ACSF (AMPA/NMDA ratios, NMDAR pharmacology), MgSO_4_ was replaced with K_2_SO_4_. Stimulating electrodes, 1–2 MΩ glass pipettes filled with ACSF, were placed in the fiber tract at the lateral edge of the LSO and stimuli were delivered via a Master 8 with Iso-Flex SIU. Cells in the higher frequency (medial and middle) limbs of the LSO were targeted using DIC-IR to identify principal cells based on their bipolar morphology and their orientation relative to the mediolateral axis of the nucleus.

Electrodes for whole-cell voltage clamp (8250 borosilicate glass, AM Systems) had resistances of 3–6 MΩ and were filled with a Cs-gluconate solution (pH 7.2) containing (in mM): 64 D-gluconic acid, 64 CsOH, 11 EGTA, 56 CsCl, 1 MgCl2•6H2O, 1 CaCl2, 10 HEPES, 0.3 GTP-Na, 4 ATPMg•3.5H2O, 0.1 mM spermine (Acros Organics). In most cases, the internal solution also contained 0.5% biocytin for subsequent histological verification of cell type. Internal solutions for some recordings of metabotropic glutamate receptors contained 100 K-gluconate, 20 KCl, 10 Na2-phosphocreatine, 10 HEPES, 0.3 GTP-Na, 4 ATP-Mg•3.5H20 (pH 7.3). Recordings (MultiClamp 700B amplifier with pClamp 10 and Axopatch 200B amplifier with pClamp 9.2; Molecular Devices) were sampled at 5 or 10 kHz, filtered at 5 kHz and saved for offline analysis with custom Matlab software. Recordings were compensated by a minimum of 80% with <10 µs lag, and were discarded if series resistance changed by more than 15% from its initial value. All recordings, except for those addressing AMPAR rectification, were made at a holding potential of −60 mV. For AMPA/NMDA ratios, pharmacology, and probability of release experiments, electrical stimulation intensity was set near a minimum intensity that reliably elicited responses and stimulation intensity remained unchanged throughout the experiment; for older cells this likely resulted in stimulation of a single input fiber. To construct input-output curves (I/O curves), initial stimulation intensities were set near zero and stimulation intensity was gradually increased until maximal response was observed (plateau in response amplitude). For minimal stimulation experiments, >150 responses were collected to stimulation that produced a response ∼40% of the time, and stimulus strength was adjusted online to maintain a failure rate of >50%. D-APV (Ascent Scientific, Tocris) and CNQX (Tocris) were added to the ACSF perfusate to block NMDARs and AMPARs. To assess AMPA/NMDA ratios, the NMDAR component (in a few cases the AMPAR component) was pharmacologically isolated using CNQX (D-APV for AMPAR), and the peak NMDAR response was measured. The mean isolated NMDAR trace was subtracted from the mean mixed glutamatergic current to obtain the mean AMPAR response, from which peak AMPAR response was measured. Ifenprodil (Ascent), a selective antagonist against GluN2B-containing NMDARs, was used to evaluate NMDAR subunit composition pharmacologically. The presence of GluR2-lacking AMPARs was assessed both by degree of rectification, assessed from recordings at −60 mV, −40 mV, 0 mV, +40 mV, and +60 mV, and by pharmacology, assessed by bath application of the selective GluR2-lacking AMPAR antagonist IEM 1460 (Tocris). In paired-pulse experiments cyclothiazide (Ascent) was included in the perfusate to prevent AMPAR desensitization. Drug concentrations used in the perfusate were: DAPV, 50 µM; CNQX, 5 µM; ifenprodil, 10 µM; IEM 1460, 100 µM; cyclothiazide, 100 µM.

To obtain minimal response amplitudes for P1-2 cells, peak current amplitude within the expected temporal window was obtained, and then all “responses” were matched to an average minimal response trace. All “responses” judged not to match the template were discarded as noise, and the remaining responses were averaged to determine minimum response strength. The rectification index RI was defined here as RI = [(I_+40mV_/(V_+40_−V_rev_)]/[(I_−60mV_/(V_−60_−V_rev_)]. Decay time constants were obtained from pharmacologically isolated NMDAR-mediated currents and were calculated as the time for the response amplitude to decay to 37% of peak. Probability of release was examined by delivering 10 pulses of electrical stimulation at 10, 20, 50, and 100 Hz. The peak current amplitudes resulting from the second to tenth pulses were divided by the peak current amplitude resulting from the first pulse (In/Ii) to determine paired-pulse ratios; peak current was measured relative to current immediately before the stimulus for each pulse. Data were analyzed using MiniAnalysis (Synaptosoft) or custom programs in Matlab (Mathworks). In general, for post-hoc statistical tests, results of significant tests (p<0.05) only are provided.

## Results

Using whole-cell voltage-clamp in acute brainstem slices from rats age P1-12, we recorded from 143 principal cells of the LSO to examine developmental changes in input number and strength, functional expression of post-synaptic receptors, and release properties of the presynaptic terminal, for the glutamatergic VCN inputs to LSO principal neurons. All recordings were made in the presence of picrotoxin (50 µM) and strychnine (1 µM) to ensure that only the isolated glutamatergic components were recorded.

### Refinement of the VCN-LSO pathway occurs during a temporal window that parallels refinement of the MNTB-LSO pathway

**Figure 1:**
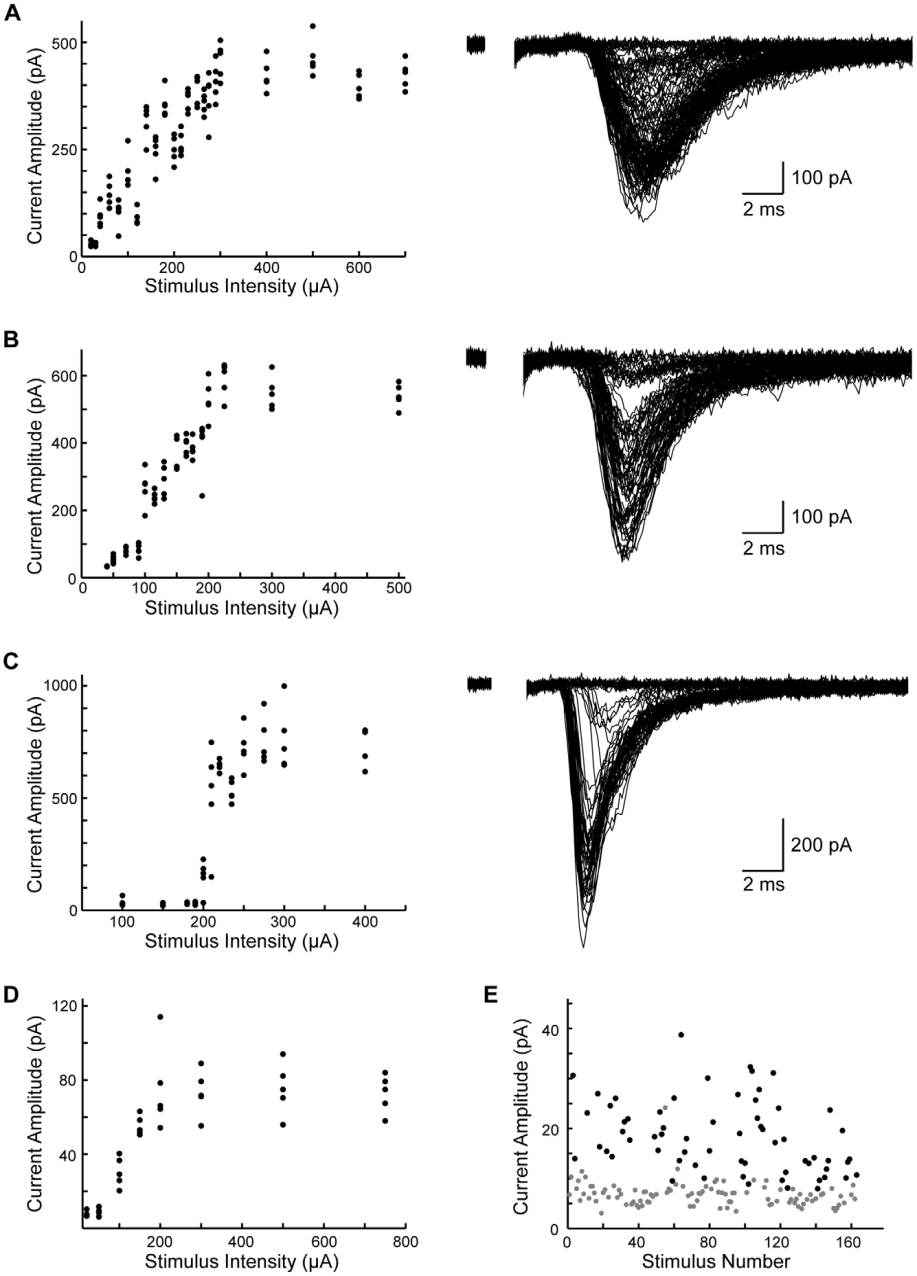
Representative input-output curves between P1 and P9. **A**: P2 cell: example individual traces and input-output curve in the VCN-LSO pathway. As shown here, input-output curves obtained for the youngest ages typically lacked clear steps. Higher stimulus amplitudes elicited no increase in response size, and have been removed for clarity. **B**: P5 cell: example individual traces and input-output curve. Stepwise increases in response amplitude could typically be distinguished at this age. Higher stimulus amplitudes elicited no increase in response size, and have been removed for clarity. **C**: P9 cell: example individual traces and input-output curve. Stepwise increases in current amplitude were nearly always distinguishable, with the number of steps rarely exceeding 3. Higher stimulus amplitudes elicited no increase in response size, and have been removed for clarity. **D**: Representative I/O-curve of P1 cell, showing average maximal response in plateau phase of 73.8±3.0 pA. In order to focus on the plateau phase, lower stimulation intensities were sparsely sampled in the youngest cells and thus the appearance of discrete steps in this example is an artifact of sparse sampling. **E**: Response amplitudes to minimal stimulation for the cell shown in D (mean minimal response of 18.3±0.9 pA). Black circles indicate “signal” responses whose shape matched an average minimal-response template; “noise” responses (gray) are shown for comparison.

In order to understand whether and how input number and strength change before hearing onset in the VCN-LSO pathway, we recorded responses to stimuli of increasing amplitude in brainstem slices from animals P1-12 and then plotted input-output curves (I/O curves) of response amplitude as a function of stimulus amplitude for each neuron. In theory, at the weakest stimulation strengths no fibers are activated, whereas stimuli of increasing strength successively recruit additional fibers, the activation of which can be seen as discrete increases in response amplitude. We found that I/O-curves from the youngest slices differed qualitatively from those obtained from slices around hearing onset. As shown in the example figure, most of the I/O curves from slices younger than P3 rose smoothly with increasing stimulus intensity, as expected for neurons that receive many weak inputs (Fig. 1A). In most of the neurons from P4-5 slices, discrete steps were apparent in the I/O relationships (Fig. 1B), and by P9 most of the I/O-curves exhibited only 1–3 steps (Fig. 1C). As shown in these representative examples, some of the steps in response amplitude at P9 were as large as the maximal responses seen before P3 (Compare the P2 example, Fig. 1A, with average maximal amplitude 427.8±9.2 pA to the P9 example, Fig. 1C, with average step sizes 174.1±15.0 pA, 418.1±21.8 pA, 153.2±22.8 pA. Note different y-axis scales.). Thus, single fibers at the older ages were able to drive LSO neurons as strongly as did the entire projection in slices of the youngest ages. Because steps in response amplitude were rare in the I/O curves of the youngest cells, it was generally not possible to directly determine the number of inputs to a single LSO cell from the I/O curves alone. In order to estimate this number for the youngest ages, we divided the average maximal response obtained from the plateau phase of the I/O-curve (Fig. 1D) by the mean observable single-fiber strength obtained from minimal stimulation (Fig. 1E) (for this P1 example cell, average maximal response = 73.8±3.0 pA and average minimal response = 18.3±0.9 pA; therefore, estimated #inputs = 4). For these youngest cells, responses were sparsely sampled at the lower stimulus intensities in order to focus on the plateau phase; thus, the appearance of steps in this example is a sampling artifact.

**Figure 2:**
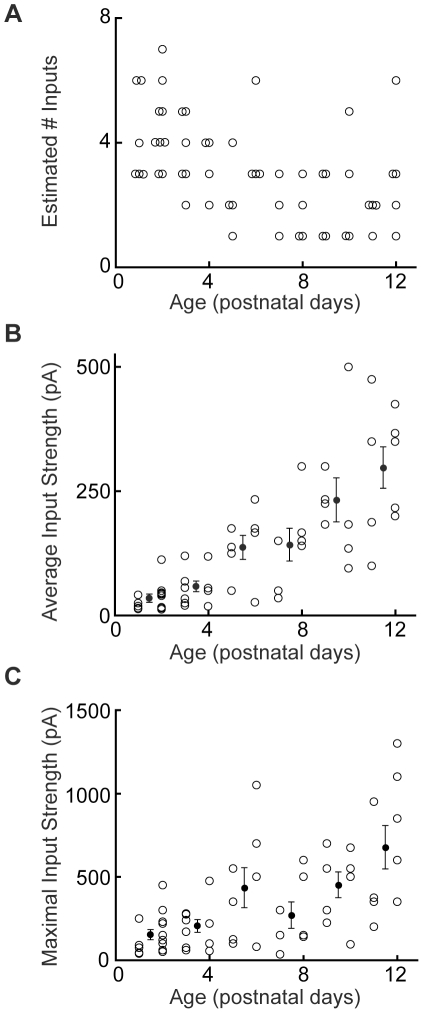
Input number and strength changes with age. **A**: Number of estimated inputs, as a function of age, for 58 LSO principal cells. On average, cells in the youngest slices receive more VCN inputs than do cells from slices around hearing onset. **B**: Mean response to stimulation of the ventral acoustic stria increased by a factor of 9 between P1/2 and P11/12 (p = 2.1 *10^−7^, Kruskal-Wallis; P3/4 vs P5/6 p = 0.013; P7/8 vs P11/12 p = 0.011, Mann-Whitney). Filled circles represent means ± SEMs at two-day intervals. **C**: Mean maximal response to VCN input increased by a factor of 4.5 between P1/2 and P11/12 (maximal input strength: p = 5.0*10^−4^, Kruskal-Wallis; P1/2 vs P11/12 p = 4.3*10^−4^; P3/4 vs P9/10 p = 0.011, Mann-Whitney).

Estimated number of inputs, mean input strength, and maximal input strength all varied as a function of age in LSO principal cells. Significantly, neurons from the youngest slices were in a distinctly different population from those in the oldest slices: before P3, 67% of all cells (10/15) received more than 3 VCN inputs, whereas from P7 onward less than 10% of all cells (2/24) had more than 3 inputs. Furthermore, before P3 no cells received fewer than 3 inputs, whereas from P7 onward 63% of all cells (15/24) received fewer than 3 inputs (Fig. 2A). For statistical testing, we assigned age groups of P1-3, P4-8 and P9-12, ages corresponding to the periods before, during, and after MNTB-LSO functional refinement, and found statistically significant differences for nearly all comparisons (Kruskal-Wallis for P1-3 vs P4-8 vs P9-12: p = 1.4*10^−4^; posthoc Mann-Whitney tests: P1-3 vs P4-12 p = 3.2*10^−5^; P1-8 vs P9-12 p = 0.0073; P1-3 vs P9-12 p = 2.54*10^−4^; P1-3 vs P4-8 p = 7.0*10^−4^; P4-8 vs P9-12 p = 0.39). At P1-2, the mean single-fiber strength was 33.9±8.2 pA and the mean maximal response was 150.3±30.3 pA, suggesting that each LSO principal cell received about 5 VCN inputs. Mean single-fiber strength increased significantly before hearing onset (p = 2.1*10^−7^; Kruskal-Wallis). By P9/10, the mean single-fiber strength had increased over five-fold to 231.9±44.2 pA and the mean maximal response to 449.4±77.3 pA, suggesting that each principal cell received only about 2 inputs (Fig. 2B,C). On average, the response to single-fiber stimulation at P9-10 was as big as the largest response that could be obtained in P1-4 slices (maximal response strength for P1-4 172.3±23.9 pA). These data are consistent with the functional elimination of immature synapses in the VCN-LSO pathway between about P3/4 and P8/9 and with an overall strengthening of those synapses that remain (mean input strength: P1-2, 33.9±8.2 pA, n = 15; P3-4, 57.7±10.7 pA, n = 11; P5-6, 136.1±24.2 pA, n = 8; P7-8, 141.7±33.0 pA, n = 7; P9-10, 231.9±44.2 pA, n = 8; P11-12, 296.8±41.7 pA, n = 9; maximal input strength: P1-2, 150.3±30.3 pA, n = 15; P3-4, 202.3±38.4 pA, n = 11; P5-6, 431.9±120.0 pA, n = 8; P7-8, 267.9±79.2 pA, n = 7; P9-10, 449.4±77.3 pA, n = 8; P11-12, 675.0±129.6 pA, n = 8). Interestingly, although elimination was apparently complete by about P8, further strengthening of VCN-LSO inputs occurred at least until hearing onset at P12 (mean input strength: p = 2.1*10^−7^, Kruskal-Wallis; P3/4 vs P5/6, p = 0.013; P7/8 vs P11/12, p = 0.011, Mann-Whitney; maximal input strength: p = 5.0*10^−4^, Kruskal-Wallis; P1/2 vs P11/12, p = 4.3*10^−4^; P3/4 vs P9/10, p = 0.011, Mann-Whitney).

### AMPAR/NMDAR ratio increases between birth and hearing onset

**Figure 3:**
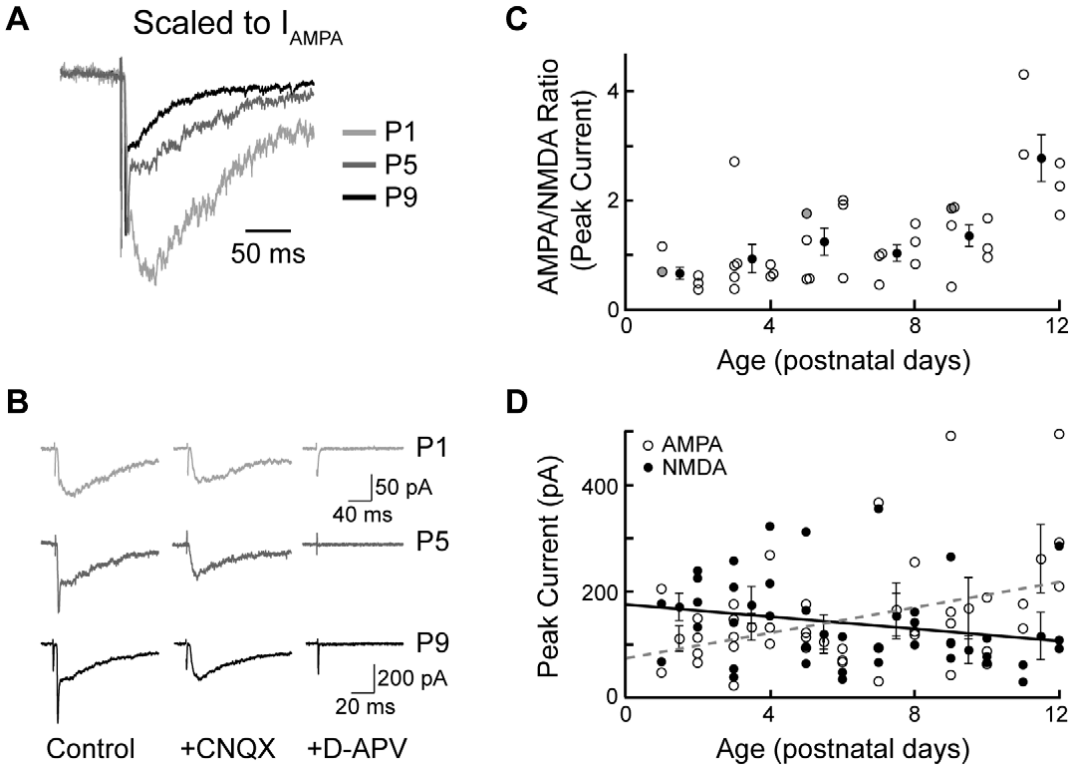
Fractional current mediated by AMPARs increases between birth and hearing onset. **A**: Representative mixed glutamatergic responses from P1, P5 and P9 neurons (average of 10 recordings), scaled to peak AMPA current; distinct AMPA and NMDA components visible in each trace. **B**: Same traces as in A, showing the mixed glutamatergic current, the pharmacologically isolated NMDAR component, and the response after application of AMPAR and NMDAR antagonists, D-APV and CNQX, which abolishes the glutamatergic response. All recordings in Mg^++^-free ACSF. Note change in scalebars for P9 recordings. **C**: Ratios of peak AMPA/NMDA current in 39 cells from slices P1-P12. During this period, AMPA/NMDA ratio increases as a function of age (p = 0.012, Kruskal-Wallis; P1/2 vs P9/10, p = 0.048; P7/8 vs P9/10, p = 0.30; P9/10 vs P11/12 p = 0.010; linear regression slope 0.14/day, r^2^ = 0.32; exponential fit r^2^ = 0.36). Filled black circles represent means ± SEMs at two-day intervals. Filled gray circles represent cells shown in A,B. **D**: Increase in AMPA/NMDA ratio with age is accompanied by small increases in average AMPA current (open circles, regressed to gray line) and decreases in average NMDAR current (filled circles, regressed to black line).

Increases in synaptic strength at many glutamatergic synapses result from the insertion of AMPARs in the postsynaptic membrane, which can be seen as an increase in the ratio of AMPAR/NMDAR peak current [29]. Thus, we examined the relative contribution of NMDARs and AMPARs to stimulation in the VCN-LSO pathway before hearing onset. To minimize possible confounds from incomplete space clamp in these large cells and potential underestimation of NMDAR currents, these experiments were performed in Mg^++^-free ACSF. Clearly visible in all recordings before the addition of receptor subtype-specific antagonists (Fig. 3A, top) were an early, fast AMPAR-mediated peak and a later, more slowly decaying NMDAR-mediated peak. Shown in Figure 3B, for the cells in Figure 3A, are the pharmacologically isolated NMDAR components from which fractional contributions to peak current were measured. Because the mixed current at all ages was carried solely by AMPA and NMDA receptors, the AMPAR contribution could be calculated by subtracting the NMDAR-mediated current from the mixed glutamatergic current. These values were used to compute the AMPA/NMDA ratio, which increased as a function of age during the postnatal period before hearing onset (Fig 3C). In the youngest cells, NMDARs contributed a larger share to the EPSC than did AMPARs, whereas by hearing onset AMPARs contributed almost three times as much current as NMDARs. (AMPA/NMDA peak current ratio P1-P2, 0.72±0.11, n = 6; P3-P4, 0.97±0.23, n = 8; P5-P6, 1.24±0.25, n = 7; P7-P8, 1.03±0.15, n = 6; P9-P10, 1.35±0.20, n = 7; P11-12, 2.77±0.43, n = 5). This increase in the AMPA/NMDA ratio could be due to an addition of AMPARs or to a loss of NMDARs or both; linear regression for each current amplitude as function of age (Fig. 3D) offered weak support for an increase in size of the AMPAR-mediated response (slope 9.9 pA/day, r^2^ 0.10), accompanied by a decrease in size of the NMDAR-mediated response (slope-7.5 pA/day, r^2^ 0.072).

### A GluN2B subunit component is prevalent before P8

**Figure 4:**
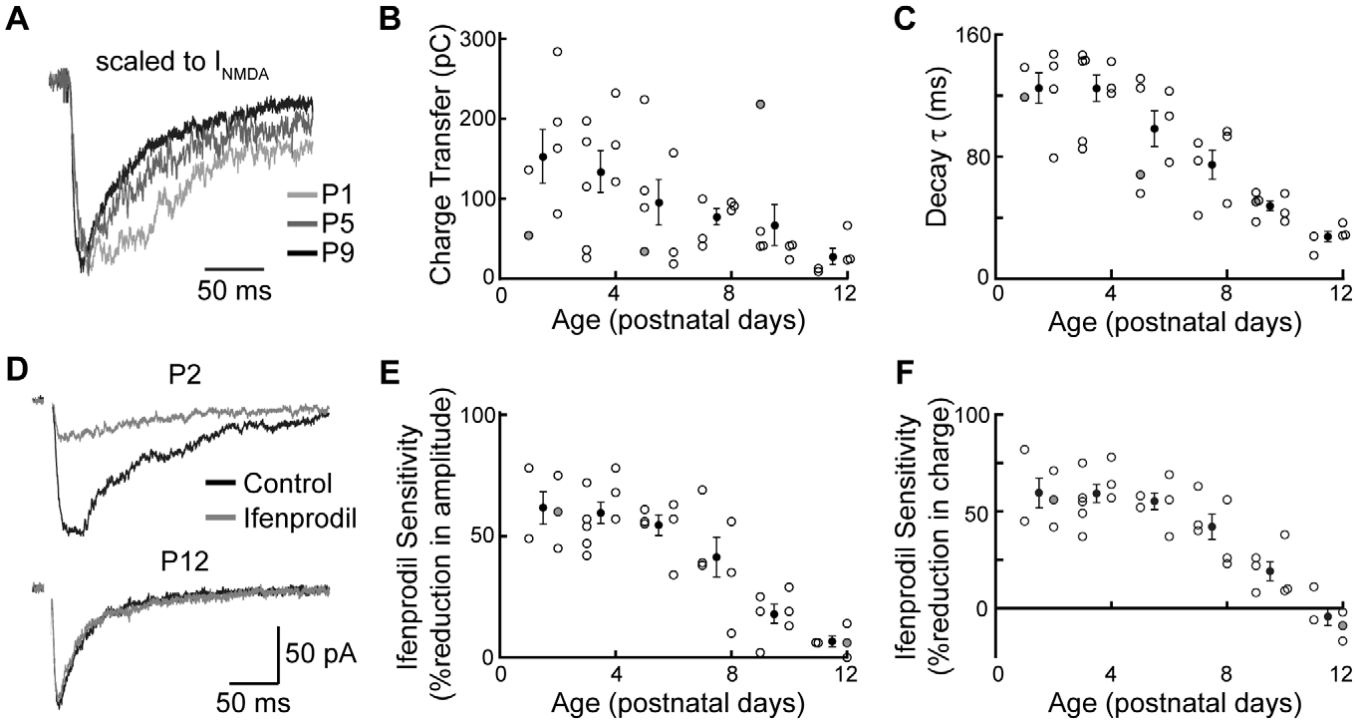
NMDAR kinetics change between P5 and P12 due to loss of GluN2B subunits. **A**: Pharmacologically isolated responses from P1, P5 and P9 neurons (same responses shown in Fig 3), scaled to peak NMDA current, overlay. Note that though these responses are somewhat unrepresentative for charge transfer (gray symbols in B and C), a change in kinetics is nevertheless apparent. **B**: NMDAR-mediated charge transfer decreases between birth and hearing onset (open circles represent individual cells; filled circles represent means ± SEMs at two-day intervals. **C**: NMDAR-mediated current decay times as a function of age. Decay times decrease rapidly after the end of the first postnatal week (p = 2.9*10^−5^, Kruskal-Wallis; P5-6 vs P7-8, p = 0.23 P7-8 vs P9-10, p = 0.10; P9-10 vs P11-12, p = 0.0025, Mann-Whitney). **D**: Median ifenprodil sensitivity from a P2 and P12 neuron; NMDAR-mediated responses in control (black) and in presence of the GluN2B-preferring antagonist ifenprodil (gray). **E**: Sensitivity to ifenprodil (10 µM), measured as % reduction from baseline response amplitude, decreases significantly at the end of the first postnatal week (p = 2.5*10^−4^, Kruskal-Wallis; P5-6 vs P7-8, p = 0.33; P7-8 vs P9-10, p = 0.037; P9-10 vs P11-12, p = 0.082, Mann-Whitney). Filled black circles indicate means in 2-day groups; filled gray circles correspond to the cells shown in D. **F**: Sensitivity to ifenprodil (10 µM), shown as % reduction from baseline charge transfer, decreases significantly at the end of the first postnatal week (p = 2.7*10−4, Kruskal-Wallis; P5-6 vs P7-8, p = 0.19; P7-8 vs P9-10, p = 0.017, P9-10 vs P11-12, p = 0.030, Mann-Whitney).

At many glutamatergic synapses, developmental plasticity is mediated by activation of NMDARs (for review, see [30]), and the expression of specific NMDAR subunits may constrain both the magnitude and phenotype of synaptic plasticity available to the synapse [31]. We asked whether the decrease in NMDAR contribution to peak current with age was due solely to a decrease in NMDAR peak current or whether it was accompanied by a change in receptor kinetics that might point to a developmental change in subunit composition. All experiments on NMDAR composition were performed in Mg^++^-free ACSF. As seen in the representative traces, the NMDAR component decayed faster at older than at younger ages (Fig. 4A). Additionally, mean charge transfer through NMDARs decreased by a factor of 5 between birth and hearing onset (Fig. 4B; P1-2, 152.2±33.9 pC, n = 6; P3-4, 133.1±26.0 pC, n = 8; P5-6, 94.8±28.6 pC, n = 7; P7-8, 76.8±10.2 pC, n = 6; P9-10, 66.2±25.6 pC, n = 7; P11-12, 26.8±10.3 pC, n = 5).

To examine more closely the change in charge transfer with age, we plotted decay time constants for the NMDAR-mediated currents as a function of age. Decay time constants, which at P1 were larger than 100 ms, by hearing onset had decreased by a factor of 4.5 (Fig. 4C; P1-2, 125.1±10.1 ms, n = 6; P3-4, 125.0±8.7 ms, n = 8; P5-6, 98.3±11.7 ms, n = 7; P7-8, 74.7±9.7 ms, n = 6; P9-10, 47.5±3.1 ms, n = 7; P11-12, 27.3±3.4 ms, n = 5). Decay time constants of order 100 ms are seen for recombinant diheteromers of both GluN1/GluN2B and GluN1/GluN2C [32]. In order to determine which of these GluN2 subunits was likely responsible for the slower decay kinetics seen before P8, we applied the GluN2B-selective antagonist ifenprodil (10 µM) while recording from cells of different ages. As seen in the representative recordings, ifenprodil affected both peak amplitude and kinetics of the NMDAR-mediated response in the first postnatal week, but had little effect thereafter (Fig. 4D). On average, bath application of ifenprodil before P8 reduced both NMDAR-mediated peak current amplitude (Fig. 4E) and charge transfer (Fig. 4F) by over 50%. In P11-12 neurons, however, ifenprodil had almost no effect on either peak current amplitude or charge transfer (% reduction of current amplitude in ifenprodil: P1-2, 61.4±6.6%, n = 5; P3-4, 59.4±4.4%, n = 8; P5-6, 54.3±4.3%, n = 6; P7-8, 41.7±8.2%, n = 6; P9-10, 17.8±3.8%, n = 6; P11-12, 6.4±2.2%, n = 5; % reduction of charge transfer in ifenprodil: P1-2, 59.2±7.6%; P3-4, 59.0±4.7%; P5-6, 55.0±4.3%; P7-8, 41.8±6.5%; P9-10, 18.8±4.9%; P11-12, −4.6±4.6%). These findings indicate that maturation of synaptic currents in the VCN-LSO pathway is due not solely to an increase in the ratio of AMPARs to NMDARs, but also to a subunit switch in the NMDARs at the end of the first postnatal week.

### EPSCs are not mediated by GluA2-lacking AMPA receptors or by metabotropic glutamate receptors

**Figure 5:**
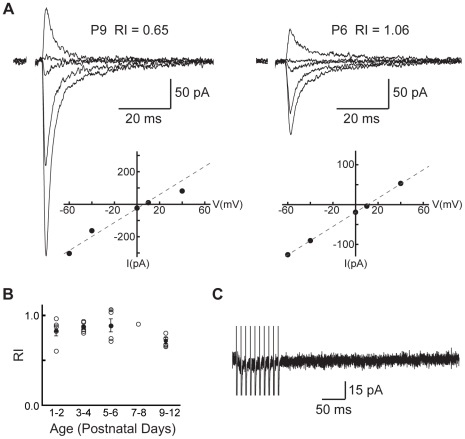
AMPARs contain GluA2 subunits. **A**: Example AMPAR responses at various holding potentials for cells with low (left) and high (right) rectification indices. Rectification indices indicate that AMPARs contain GluA2 subunits during the period of VCN-LSO input functional refinement (n = 21). **B**: Rectification indices of pharmacologically isolated AMPAR responses (average RI = 0.83±0.03, n = 21, P1-12). Symbols corresponding to cells shown in A are shaded. **C**: Representative recording from a cell (P1) in response to high-frequency stimulation in an attempt to activate mGluRs. With CNQX and APV in the perfusate, stimulation at 100 Hz failed to elicit mGluR-mediated currents in any cell thus examined (n = 21).

We asked whether other glutamate receptors known to mediate synaptic plasticity, such as either the GluA2-lacking, calcium-permeable AMPARs (CP-AMPARs) or the metabotropic glutamate receptors (mGluRs), might contribute to the EPSCs measured after stimulation of VCN-LSO fibers. After applying APV to isolate the AMPAR-mediated response, we obtained current-voltage relationships and used subunit-specific pharmacology to test for the presence of CP-AMPARs at VCN-LSO synapses (Fig. 5A). AMPARs did not exhibit substantial inward rectification at any age examined (Fig. 5B; mean RI = 0.83±0.03, n = 21). Despite an apparent trend toward lower rectification indices (RIs) immediately before hearing onset, no significant age-dependent effects were seen (Kruskal-Wallis for age effects: p = 0.13). Additionally, the CP-AMPAR specific antagonist IEM 1460 had no effect on AMPAR-mediated currents at any age (% reduction = 7.0±6.6%, n = 10; data not shown). These data agree with earlier Ca^++^-imaging results in neonatal mouse VCN-LSO showing that AMPAR-elicited Ca^++^ transients are not due to influx through CP-AMPARs [28].

We also did not observe postsynaptic currents resulting from mGluR activation. As shown in Figure 3, the EPSC to single-pulse stimulation in the ventral acoustic stria was abolished by application of CNQX and APV. With CNQX and APV in the perfusate, we further stimulated VCN afferents at 100 and/or 500 Hz, frequencies shown to activate mGluR-mediated Ca^++^-transients in the mouse VCN-LSO pathway, and we did not observe any mGluR-mediated currents when recording for 1 second or less in response to high frequency stimulation (Fig. 5C; n = 21).

### Paired-pulse depression at VCN-LSO synapses decreases between birth and hearing onset

**Figure 6:**
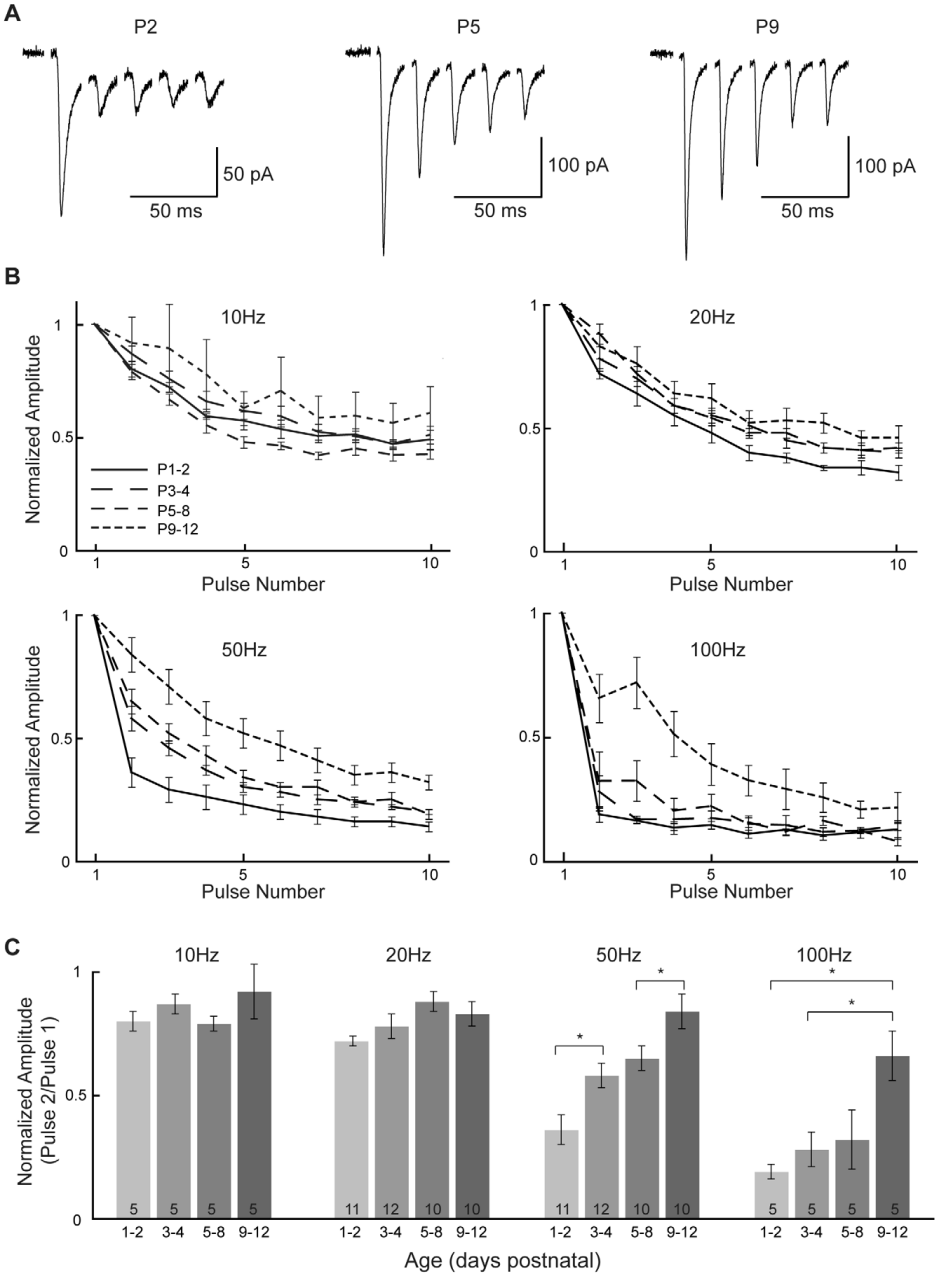
Paired-pulse depression decreases between birth and hearing onset. **A**: Representative responses to 50 Hz pulse-trains in P2, P5, and P9 cells; responses to the first five pulses only are shown. Greater paired-pulse depression between pulse 1 and 2 is evident in the youngest cell than in the older cells. **B**: Average responses to 10-pulse trains at four frequencies. At stimulation frequencies of 20 Hz or less, few differences are seen between ages. At 50 and 100 Hz, clear differences are obvious between age groups, and only the oldest (P9-12) cells are able to follow 100 Hz trains. **C**: Group data from **B**, showing paired-pulse ratios as pulse 2/pulse 1. At 10 and 20 Hz, age differences are not significant at the p<0.05 level (p = 0.48 and p = 0.063, one-way ANOVA). At 50 Hz, significant age differences are seen in paired-pulse depression (one-way ANOVA:  = 3.3*10^−5^), with greater depression in P1-2 slices than in P3-4 slices (posthoc Mann-Whitney p = 0.014) and in P5-8 slices than in P9-12 slices (p = 0.041). At 100 Hz, significant age differences are also seen (one-way ANOVA p = 0.071), with paired-pulse depression greater in P1-2 (p = 0.0018) and P3-4 slices than in P9-12 slices (p = 0.012).

Circuits in the auditory brainstem are capable of supporting among the fastest firing rates in the nervous system. Additionally, varying patterns of activity in the mouse VCN-LSO pathway have been shown to differentially activate different glutamate receptor subtypes and different calcium sources [28]. To determine the capability of VCN-LSO synapses to support repetitive activity in the developing circuit, we added cyclothiazide to the perfusate to prevent AMPAR desensitization and measured responses to 50 Hz pulse-train stimuli delivered to the ventral acoustic stria. As judged by paired-pulse depression (PPD), neurons in the youngest slices were unable to follow at 50 Hz, a relatively slow frequency for the auditory brainstem (Fig. 6A). In order to ask how frequency-following matures between P1 and P12 we delivered pulse trains of 10 stimuli at 10, 20, 50, and 100 Hz to VCN-LSO fibers. Stimulation at lower frequencies (10 and 20 Hz, or 100 and 50 ms inter-stimulus intervals) yielded similar response profiles at all ages (Fig. 6B), whereas stimulation at higher frequencies (50 and 100 Hz, or 20 and 10 ms inter-stimulus intervals) revealed distinctly different profiles for the different age groups (50 Hz: p = 3.3*10^−5^; 100 Hz: p = 0.071, one-way ANOVA). PPD at 50 Hz (Fig. 6C) was significantly larger in P1-2 than in P3-4 cells (p = 0.014) and in P5-8 than in P9-12 cells (p = 0.041; [Paired-pulse ratios at 50 Hz: P1-P2, 0.36±0.06; P3-P4, 0.58±0.05; P5-P8, 0.65±0.05; P9-P12, 0.84±0.07]). At 100 Hz, PPD was also significantly larger in P1-2 and in P3-4 cells than in P9-12 cells (p = 0.0018; p = 0.012; [Paired-pulse ratios at 100 Hz: P1-P2, 0.19±0.03; P3-P4, 0.28±0.07; P5-P8, 0.32±0.12; P9-P12, 0.66±0.10]). These data suggest that changes in probability of neurotransmitter release, rate of vesicle recruitment, number of postsynaptic receptors, or some combination of these, follow a developmental trend that coincides with functional refinement at VCN-LSO synapses.

## Discussion

### Functional refinement in the VCN-LSO pathway parallels refinement in the MNTB-LSO pathway

Using whole-cell patch recordings in the acute slice preparation, we have examined developmental refinement in the excitatory VCN-LSO pathway. We find that the number of functional inputs decreases, and that mean and maximal input strength increase, during the first postnatal week, and thus that refinement in the VCN-LSO pathway occurs during a similar period to that in the opposing, MNTB-LSO pathway. We find that refinement in the VCN-LSO pathway is accompanied by a decrease in the NMDAR fractional contribution and an increase in the AMPAR contribution. Additionally, during the period of functional synapse elimination, much of the NMDAR-mediated current is borne by GluN2B-containing receptors, which disappear rapidly after P8. Finally, frequency-following at VCN-LSO synapses increases progressively between birth and hearing onset.

Our estimates of input number and of percentage decrease are lower than those reported for the MNTB pathway [25]. These discrepancies may reflect real differences between the two pathways or may be artifacts of slice preparation. Our data should be used cautiously for inferring exact numbers of inputs. Because in any slice preparation an unknown number of fibers is severed, precise input numbers cannot be taken from this study, which provides information about lower bounds and about relative numbers of inputs. Also, because the geometry of rat auditory brainstem precludes a viable slice containing the VCN cell bodies with their fibers that project to the LSO, we cannot apply the more elegant techniques used to demonstrate major refinement in the MNTB-LSO pathway during the period P3-8 [25]. Despite our likely underestimation of input number, clear trends are apparent: all P1-3 neurons received ≥3 VCN inputs whereas most P9-12 neurons received <3 inputs, differences supported by non-parametric ANOVA. Our results thus suggest that functional elimination in the VCN-LSO pathway occurs before P8.

Converging neural pathways can be refined either by first establishing and refining one projection and then refining the second projection to match the first, or by refining the two projections simultaneously [33]–[35]. The refinement observed here is consistent with the second model. We suggest that, like the MNTB-LSO pathway, the VCN-LSO pathway undergoes synaptic silencing during the first postnatal week, possibly using similar mechanisms, and that the excitatory and inhibitory maps are brought into tonotopic registration after hearing onset [14] ([10] for review). We note a continued strengthening in the VCN-LSO pathway between P8 and hearing onset that has not been reported in the MNTB-LSO pathway. Further strengthening after the period of synaptic silencing could use activity-dependent mechanisms proposed to operate in the first postnatal week, or could involve generalized homeostatic strengthening of the excitatory pathway to compensate for a progressive increase in driving force for hyperpolarizing inhibitory inputs between P8 and hearing onset [36]. Finally, little is known about glial cells and glutamate re-uptake in the immature LSO, and spillover to peri- or extra-synaptic NMDARs, in which the GluN2B subunit would likely predominate, could be especially prevalent at young ages [37]. If so, maturation of glutamate uptake and reduction of spillover [38] might cause an increase in AMPAR/NMDAR ratio. Alternatively, increases in AMPAR/NMDAR ratio are commonly attributed to insertion of AMPARs, which could also contribute to increases in input strength.

### Glutamate receptor subtypes in the VCN-LSO pathway change before hearing onset

In addition to similarities of time course, we observed other similarities between the MNTB and VCN inputs to LSO before hearing onset. At MNTB-LSO synapses, NMDAR-mediated signaling is high before P8, coinciding with the period of major synapse elimination in the MNTB-LSO pathway and declines thereafter as glutamate release declines [23], [25]. Furthermore, NMDAR signaling is lost just as chloride transporter expression matures and these synapses transition from depolarizing to hyperpolarizing GABA/glycinergic transmission [13], [36], [39], [40]. The finding that glutamate is released onto NMDARs at depolarizing GABA/glycinergic synapses during a period of developmental refinement has led to the hypothesis that co-release of glutamate together with depolarizing GABA/glycine activate NMDARs that in turn mediate plasticity in this immature inhibitory pathway, a model corroborated by the report that mice lacking glutamate release from MNTB terminals exhibit perturbed refinement of the MNTB-LSO pathway [24]. As in the MNTB-LSO pathway, the developmental changes in NMDAR-mediated signaling in the VCN-LSO pathway occur during a period of functional refinement and are characterized by prominent GluN2B activation that is lost after about P8 (this study; Case and Gillespie, unpublished observations). Regardless of the exact composition of NMDARs in the first postnatal week, we suggest that GluN2B is likely replaced by GluN2A, in a subunit switch similar to that seen in many other areas during development [41], [42].

The expression of specific GluN2 subunits determines receptor open probability and conductance, interactions with scaffolding proteins, kinase affinities, and mobility in the plasma membrane, among other factors [43]–[48]. The GluN2 subunits also show widely different developmental expression profiles in space and time [41], [42], and a large body of work has focused on the roles of specific GluN2 subunits, and the ratios of these subunits, in mediating developmental plasticity directly or through control of metaplasticity (reviewed in [30], [49]). Substantial debate has emerged over whether GluN2A or GluN2B may specifically mediate depression versus potentiation [31], [50]; we note that a GluN2B component is prominent during a period that includes both silencing and strengthening, whereas GluN2B is rapidly downregulated during a subsequent period of generalized strengthening. If NMDAR activation is required for developmental plasticity in this pathway, these correlations may suggest that GluN2B mediates synaptic silencing.

In addition to NMDARs, the GluA2-lacking, Ca^++^-permeable AMPARs (CP-AMPARs) and various mGluRs can also be developmentally expressed and/or can mediate plasticity at central synapses [51]-[53]. Our electrophysiological results agree with an earlier Ca^++^-imaging study in mouse [28], and we conclude that synapses in the early postnatal VCN-LSO pathway do not express CP-AMPARs. In contrast to the imaging study, we found no evidence for mGluR-initiated EPSCs in our recordings, even with high-frequency stimulation. This discrepancy could result from species differences, but a more likely cause may be that as conductances initiated by mGluR activation require release of Ca^++^ from internal stores and downstream activation of TRP channels [54], any EPSCs resulting from mGluR activation here would have occurred at latencies too long to appear in whole-cell recordings of ≤500–1000 ms.

### Paired-pulse depression declines between birth and hearing onset

We found that at P1 the VCN-LSO pathway could support synaptic transmission at 20 Hz, but not at frequencies ≥50 Hz. The ability of this pathway to support repetitive synaptic transmission increased with age, however, as has also been reported in the MNTB-LSO pathway of mouse [22]. Although the significant differences seen at 50 and 100 Hz may represent step-increases in the ability of this pathway to support repetitive firing (e.g., at the opening of the P3-8 window for refinement of the MNTB-LSO pathway and again at hearing onset), we suggest that frequency-following varies smoothly with age and stimulation frequency and that these apparent step-increases represent sampling artifacts. Possibly, the graded differences in frequency-following seen almost until hearing onset result from the staggered maturation of multiple, interacting processes, such as calcium-channel proximity, vesicle population, and vesicle recycling, among others [55], [56]. Interestingly, PPD observed in the VCN-LSO pathway was similar to that seen for glutamate, but not for GABA/glycine, release in the MNTB-LSO pathway (Case and Gillespie, unpublished observations), despite the fact that the glutamatergic vesicle populations in the two pathways likely use both different vesicular glutamate transporters and different synaptotagmins [55]. Finally, we expect the mature VCN-LSO pathway to exhibit release probabilities and frequency-following similar to that in the MNTB-LSO pathway. Estimating *in vivo* release probabilities from *in vitro* experiments can be problematic [57], and the different release properties we note may result from slight variability among slices. Nevertheless, understanding how release probability and frequency-following are related in these opposing circuits may contribute to understanding the computation performed by the mature LSO.

## References

[1] Boudreau JC, Tsuchitani C (1968). Binaural interaction in the cat superior olive S segment.. J Neurophysiol.

[2] Caird D, Klinke R (1983). Processing of binaural stimuli by cat superior olivary complex neurons.. Exp Brain Res.

[3] Tollin DJ (2009). The lateral superior olive: a functional role in sound source localization.. Neuroscientist.

[4] Cant NB, Casseday JH (1986). Projections from the anteroventral cochlear nucleus to the lateral and medial superior olivary nuclei.. J Comp Neurol.

[5] Wu SH, Kelly JB (1992). Synaptic pharmacology of the superior olivary complex studied in mouse brain slice.. J Neurosci.

[6] Moore MJ, Caspary DM (1983). Strychnine blocks binaural inhibition in lateral superior olivary neurons.. J Neurosci.

[7] Bledsoe SC, Snead CR, Helfert RH, Prasad V, Wenthold RJ (1990). Immunocytochemical and lesion studies support the hypothesis that the projection from the medial nucleus of the trapezoid body to the lateral superior olive is glycinergic.. Brain Res.

[8] Smith PH, Joris PX, Carney LH, Yin TC (1991). Projections of physiologically characterized globular bushy cell axons from the cochlear nucleus of the cat.. J Comp Neurol.

[9] Caspary DM, Finlayson PG, Altschuler RA, Bobbin RP, Clopton BM, Hoffman DW (1991). The lateral superior olive: a functional role in sound source localization.. Neurobiology of Hearing: The Central Auditory System: Lippincott.

[10] Sanes DH, Friauf E (2000). Development and influence of inhibition in the lateral superior olivary nucleus.. Hear Res.

[11] Kandler K, Clause A, Noh J (2009). Tonotopic reorganization of developing auditory brainstem circuits.. Nat Neurosci.

[12] Kandler K, Friauf E (1993). Pre- and postnatal development of efferent connections of the cochlear nucleus in the rat.. J Comp Neurol.

[13] Kandler K, Friauf E (1995). Development of glycinergic and glutamatergic synaptic transmission in the auditory brainstem of perinatal rats.. J Neurosci.

[14] Sanes DH, Rubel EW (1988). The ontogeny of inhibition and excitation in the gerbil lateral superior olive.. J Neurosci.

[15] Oertel D (1999). The role of timing in the brain stem auditory nuclei of vertebrates.. Annu Rev Physiol.

[16] Lippe WR (1994). Rhythmic spontaneous activity in the developing avian auditory system.. J Neurosci.

[17] Kros CJ, Ruppersberg JP, Rusch A (1998). Expression of a potassium current in inner hair cells during development of hearing in mice.. Nature.

[18] Beutner D, Moser T (2001). The presynaptic function of mouse cochlear inner hair cells during development of hearing.. J Neurosci.

[19] Tritsch NX, Yi E, Gale JE, Glowatzki E, Bergles DE (2007). The origin of spontaneous activity in the developing auditory system.. Nature.

[20] Tritsch NX, Bergles DE (2010). Developmental regulation of spontaneous activity in the Mammalian cochlea.. J Neurosci.

[21] Sanes DH, Takacs C (1993). Activity-dependent refinement of inhibitory connections.. Eur J Neurosci.

[22] Kim G, Kandler K (2010). Synaptic changes underlying the strengthening of GABA/glycinergic connections in the developing lateral superior olive.. Neuroscience.

[23] Gillespie DC, Kim G, Kandler K (2005). Inhibitory synapses in the developing auditory system are glutamatergic.. Nat Neurosci.

[24] Noh J, Seal RP, Garver JA, Edwards RH, Kandler K (2010). Glutamate co-release at GABA/glycinergic synapses is crucial for the refinement of an inhibitory map.. Nat Neurosci.

[25] Kim G, Kandler K (2003). Elimination and strengthening of glycinergic/GABAergic connections during tonotopic map formation.. Nat Neurosci.

[26] Kotak VC, Sanes DH (1996). Developmental influence of glycinergic transmission: regulation of NMDA receptor-mediated EPSPs.. J Neurosci.

[27] Caicedo A, Eybalin M (1999). Glutamate receptor phenotypes in the auditory brainstem and mid-brain of the developing rat.. Eur J Neurosci.

[28] Ene FA, Kullmann PH, Gillespie DC, Kandler K (2003). Glutamatergic calcium responses in the developing lateral superior olive: receptor types and their specific activation by synaptic activity patterns.. J Neurophysiol.

[29] Saal D, Dong Y, Bonci A, Malenka RC (2003). Drugs of abuse and stress trigger a common synaptic adaptation in dopamine neurons.. Neuron.

[30] Malenka RC, Bear MF (2004). LTP and LTD: an embarrassment of riches.. Neuron.

[31] Liu L, Wong TP, Pozza MF, Lingenhoehl K, Wang Y (2004). Role of NMDA receptor subtypes in governing the direction of hippocampal synaptic plasticity.. Science.

[32] Vicini S, Wang JF, Li JH, Zhu WJ, Wang YH (1998). Functional and pharmacological differences between recombinant N-methyl-D-aspartate receptors.. J Neurophysiol.

[33] Knudsen EI (2002). Instructed learning in the auditory localization pathway of the barn owl.. Nature.

[34] Triplett JW, Owens MT, Yamada J, Lemke G, Cang J (2009). Retinal input instructs alignment of visual topographic maps.. Cell.

[35] Deeg KE, Sears IB, Aizenman CD (2009). Development of multisensory convergence in the Xenopus optic tectum.. J Neurophysiol.

[36] Ehrlich I, Lohrke S, Friauf E (1999). Shift from depolarizing to hyperpolarizing glycine action in rat auditory neurones is due to age-dependent Cl- regulation.. J Physiol 520 Pt.

[37] Tovar KR, Westbrook GL (1999). The incorporation of NMDA receptors with a distinct subunit composition at nascent hippocampal synapses in vitro.. J Neurosci.

[38] Scimemi A, Tian H, Diamond JS (2009). Neuronal transporters regulate glutamate clearance, NMDA receptor activation, and synaptic plasticity in the hippocampus.. J Neurosci.

[39] Kakazu Y, Akaike N, Komiyama S, Nabekura J (1999). Regulation of intracellular chloride by cotransporters in developing lateral superior olive neurons.. J Neurosci.

[40] Kullmann PH, Kandler K (2001). Glycinergic/GABAergic synapses in the lateral superior olive are excitatory in neonatal C57Bl/6J mice.. Brain Res Dev Brain Res.

[41] Sheng M, Cummings J, Roldan LA, Jan YN, Jan LY (1994). Changing subunit composition of heteromeric NMDA receptors during development of rat cortex.. Nature.

[42] Monyer H, Burnashev N, Laurie DJ, Sakmann B, Seeburg PH (1994). Developmental and regional expression in the rat brain and functional properties of four NMDA receptors.. Neuron.

[43] Monyer H, Sprengel R, Schoepfer R, Herb A, Higuchi M (1992). Heteromeric NMDA receptors: molecular and functional distinction of subtypes.. Science.

[44] Strack S, Colbran RJ (1998). Autophosphorylation-dependent targeting of calcium/calmodulin-dependent protein kinase II by the NR2B subunit of the N-methyl- D-aspartate receptor.. J Biol Chem.

[45] Barria A, Malinow R (2005). NMDA receptor subunit composition controls synaptic plasticity by regulating binding to CaMKII.. Neuron.

[46] Cull-Candy S, Brickley S, Farrant M (2001). NMDA receptor subunits: diversity, development and disease.. Curr Opin Neurobiol.

[47] Prybylowski K, Chang K, Sans N, Kan L, Vicini S (2005). The synaptic localization of NR2B-containing NMDA receptors is controlled by interactions with PDZ proteins and AP-2.. Neuron.

[48] Groc L, Heine M, Cousins SL, Stephenson FA, Lounis B (2006). NMDA receptor surface mobility depends on NR2A-2B subunits.. Proc Natl Acad Sci U S A.

[49] Philpot BD, Cho KK, Bear MF (2007). Obligatory role of NR2A for metaplasticity in visual cortex.. Neuron.

[50] Morishita W, Lu W, Smith GB, Nicoll RA, Bear MF (2007). Activation of NR2B-containing NMDA receptors is not required for NMDA receptor-dependent long-term depression.. Neuropharmacology.

[51] Liu SQ, Cull-Candy SG (2000). Synaptic activity at calcium-permeable AMPA receptors induces a switch in receptor subtype.. Nature.

[52] Ho MT, Pelkey KA, Topolnik L, Petralia RS, Takamiya K (2007). Developmental expression of Ca2+-permeable AMPA receptors underlies depolarization-induced long-term depression at mossy fiber CA3 pyramid synapses.. J Neurosci.

[53] Oliet SH, Malenka RC, Nicoll RA (1997). Two distinct forms of long-term depression coexist in CA1 hippocampal pyramidal cells.. Neuron.

[54] Ene FA, Kalmbach A, Kandler K (2007). Metabotropic glutamate receptors in the lateral superior olive activate TRP-like channels: age- and experience-dependent regulation.. J Neurophysiol.

[55] Cooper AP, Gillespie DC (2011). Synaptotagmins I and II in the developing rat auditory brainstem: Synaptotagmin I is transiently expressed in glutamate-releasing immature inhibitory terminals.. J Comp Neurol.

[56] Wang LY, Neher E, Taschenberger H (2008). Synaptic vesicles in mature calyx of Held synapses sense higher nanodomain calcium concentrations during action potential-evoked glutamate release.. J Neurosci.

[57] Lorteije JA, Rusu SI, Kushmerick C, Borst JG (2009). Reliability and precision of the mouse calyx of Held synapse.. J Neurosci.

